# Higher dietary magnesium intake is associated with lower body mass index, waist circumference and serum glucose in Mexican adults

**DOI:** 10.1186/s12937-018-0422-2

**Published:** 2018-12-05

**Authors:** Analí Castellanos-Gutiérrez, Tania G. Sánchez-Pimienta, Alicia Carriquiry, Teresa H. M. da Costa, Ana Carolina Ariza

**Affiliations:** 1 Center for Nutrition and Health Research, National Institute of Public Health, Mexico. Av. Universidad 655, Col. Santa María Ahuacatitlán. Cerrada los pinos y caminera. C.P, 62100 Cuernavaca, Morelos Mexico; 2 Department of Statistics, Iowa State University, Ames, Iowa 50011 USA; 3 Department of Nutrition, School of Health Science, University of Brasilia, Brasilia - DF, CEP 70910-900 Brazil; 4 National Council of Science and Technology (CONACYT) - Center for Nutrition and Health Research, National Institute of Public Health, Cuernavaca, Morelos Mexico

**Keywords:** Obesity, Magnesium, Glucose, Mexico, Adult

## Abstract

**Background:**

Obesity and diabetes mellitus (DM) are public health concerns in Mexico of top-level priority due to their high prevalence and their growth rate in recent decades. The accumulation of adipose tissue leads to an unbalanced release of pro-oxidant factors, which causes cellular damage and favors the development of comorbidities. Recent evidence suggests that oxidative stress also promotes the accumulation of adipose tissue and the development of insulin resistance. The objective of this study is to evaluate the association between usual intake of antioxidant nutrients, specifically vitamins A, C, E and magnesium with body mass index (BMI), waist circumference (WC) and serum glucose concentrations in a representative sample of Mexican adults.

**Methodology:**

We analyzed data on diet, BMI, WC and serum glucose from the Mexican National Health and Nutrition Survey 2012. Analysis included 20- to 65-year-old adults without a known diagnosis of DM (*n* = 1573). Dietary information was obtained using the five-step multiple-pass method developed by the United States Department of Agriculture and adapted to the Mexican context. Nutrient usual intake distributions were estimated using the Iowa State University method, through the “Software for Intake Distribution Estimation” (PC-Side) v.1.02. Associations were analyzed using multivariate regression models.

**Results:**

Higher dietary magnesium intake was associated with lower markers of adiposity, so that an increase in 10 mg per 1000 kcal/day of magnesium was associated with an average decrease in BMI of 0.72% (95% CI: -1.36, − 0.08) and 0.49 cm (95% CI: -0.92, − 0.07) of WC. Additionally, in women with normal glucose concentrations, an increase in magnesium intake was associated with an average decrease in serum glucose by 0.59% (95% CI: -1.08, − 0.09).

**Conclusion:**

The results suggest that magnesium intake is associated with lower BMI, WC and serum glucose in Mexican population. However, more studies are required to elucidate the nature of this association.

**Electronic supplementary material:**

The online version of this article (10.1186/s12937-018-0422-2) contains supplementary material, which is available to authorized users.

## Background

Obesity and its comorbidities, including diabetes mellitus (DM), are public health concerns worldwide. In the last decades, they received top-level priority due to their high prevalence and speed of growth [1]. In Mexico, in the year 2016, the estimated prevalence of overweight and obesity in adults was 72.5%, showing a marked increase from a prevalence of 56% in the year 2000 [2, 3]. Furthermore, unpublished data estimated that in 2012, 22% of the Mexican adult population were glucose intolerant, and 13% of women and 14% of men had DM [S. Villalpando, personal communication, January 2018].

The accumulation of adipose tissue favors the presence of systemic oxidative stress (OS), which can be a trigger for the development of alterations in glucose metabolism [4–8]. However, recent evidence suggests that this mechanism may also occur inversely, where OS can favor the accumulation of adipose tissue. It has been observed that treatment with antioxidant nutrients prevents in vitro formation of adipocytes from pre-adipocytes [9], and in animal models prevents the excessive accumulation of adipose tissue associated with diet [10, 11]. In addition, OS causes a disruption in the production of cytokines [4], which together with a high production of reactive oxygen species (ROS), decreases glucose uptake in muscular and adipose tissue [6, 7] and decreases insulin secretion in pancreatic beta cells [8].

Antioxidant nutrients mitigate the damage caused by OS through different mechanisms. Vitamin A inhibits lipid peroxidation by trapping free radicals in cell membranes [12], regulates leptin mRNA in adipose tissue and also exerts an inhibitory effect on adipogenesis [13]. Vitamin C is a redox catalyst that neutralizes ROS [14], while vitamin E in its alfa-tocopherol form reacts with ROS produced in lipid peroxidation, protecting the cell membranes [15, 16]. Magnesium is involved in energy metabolism since intracellular adenosine triphosphate (ATP) is in the form of ATP-Mg [17] and it is also a cofactor of several antioxidant enzymes, including superoxide dismutase (SOD), one of the most important antioxidant enzymes [17, 18].

According to the results from the 2012 Mexican National Health and Nutrition Survey (ENSANUT) there is a high prevalence of inadequate intake of several nutrients with antioxidant function including vitamin A, C, E and magnesium, in the adult Mexican population [19, 20]. However, this prevalence has not been characterized by body weight status or serum glucose concentrations in this population. Additionally, since previous studies have shown a relationship between antioxidant nutrients intake, adiposity and insulin resistance [10, 21–26], the objective of this study is to evaluate the association between dietary intake of vitamins A, C, E, magnesium, and body mass index (BMI), waist circumference (WC), and serum glucose concentrations in a representative sample of 20- to 65-year-old Mexican adults.

## Methods

### Study design

The ENSANUT 2012 is a population-based multistage probabilistic survey representative of the Mexican population at a national, regional and state level, for urban and rural areas. The main objective of the survey was to quantify the distribution of health and nutrition parameters among the Mexican population as well as their determinants. Information from 96,031 individuals belonging to 50,528 randomly selected households was collected by face-to-face interviews carried out between October 2011 and May 2012. The complete methodology has been previously described elsewhere [27].

### Participants

For this study, we considered 20- to 65-year-old nonpregnant nonlactating participants with diet, serum glucose, and anthropometric information (*n* = 1739). Pregnant or lactating women were not included because their nutritional needs and body composition are different from the rest of the women. We excluded from the analysis subjects with low weight (*n* = 14), defined as a BMI < 18.5 kg/m^2^, or with known diagnosis of DM (*n* = 152). The final study sample included 1573 participants. Reporting was conducted in accordance with the STROBE statement [see Additional file 1].

### Diet

Detailed diet information was obtained with a 24-h dietary recall applied by trained personnel to a randomly selected subsample of ∼11% (*n* = 10,886) of the total respondents of the ENSANUT 2012 using the five-step multiple-pass method developed by the United States Department of Agriculture (USDA) [28] and adapted to the Mexican context [29]. A second 24-h recall was applied to a ∼ 9% (*n* = 981) random subsample. For this study, 9.3% (*n* = 162) of the 1573 participants answered a second 24-h recall. Interviews were performed between Monday and Sunday, and repeated measurements were obtained on non-consecutive days with an average of 2.4 (± 1.2) days between each other. The detailed methodology for the collection of dietary information is described elsewhere [29].

Intake of vitamins A, C, E and magnesium were estimated using the food composition table compiled by the National Institute of Public Health (INSP) of Mexico [30]. Nutrient intake values higher than 1.5 times the 99th percentile were considered excessive, for vitamin A (*n* = 8), vitamin C (*n* = 9), vitamin E (*n* = 4), and magnesium (*n* = 3), and were replaced with random values between the 95th percentile and 1.5 times de 99th percentile of a uniform distribution.

### Anthropometric measurements

Information on weight, height and waist circumference measurements were obtained by trained and standardized personnel using conventional and internationally accepted protocols [31, 32]. Height values between 1.3 and 2.0 m, and BMI values below 58 kg/m^2^ were considered as valid data. All observations in the eligible sample were valid data. BMI categorization was based on the criterion proposed by the World Health Organization (WHO) [33]. The WC cut-off points used are the ones proposed by the International Diabetes Federation (IDF) (94 cm in men and 80 cm in women) [34].

### Serum glucose

Serum glucose concentrations were analyzed in a sample of venous blood drawn from the antecubital vein and obtained after a minimum 8-h fast. It was collected in tubes without anticoagulant and was centrifuged in situ. Samples were stored in cryovials, placed in liquid nitrogen and stored at − 70 °C until assay. Measurements were performed at the laboratory of the Center for Nutrition and Health Research, INSP using an automatized glucose oxidase method performed in an Abbot ARCHITECT Analyzer.

Categorization was performed using the cut-off points proposed by the American Diabetes Association (ADA), which establish that concentrations between 40 and < 100 mg/dL are normal, 100 to < 126 mg/dL indicate glucose intolerance, and ≥ 126 mg/dL indicate DM [35]. To improve precision on the estimates for the prevalence of inadequate intake of nutrients, we combined categories of glucose intolerance and DM into one category of impaired glucose intolerance (serum glucose ≥100 mg/dL).

### Physical activity

Physical activity was measured using the validated short form of the International Physical Activity Questionnaire (IPAQ-SF), which includes 9 items that collect information about frequency, duration and intensity of physical activity [36, 37]. An individual estimate of minutes per week of moderate physical activity was obtained adding the reported time spent doing moderate activity and two times the reported time doing vigorous activity. Based on the WHO recommendations for physical activity [38], participants were categorized as active (≥ 300 min per week), moderately active (150 to < 300), and inactive (< 150 min per week).

### Statistical analysis

Nutrient usual intake distributions were estimated using the Iowa State University (ISU) method, through the “Software for Intake Distribution Estimation” (PC-Side) v.1.02. This method eliminates the effect of day-to-day variability in intake of each person, which results in a more accurate estimate of the prevalence of nutrient inadequacy. Intakes below the estimated average requirement (EAR) [39, 40] established by the Institute of Medicine of the United States [41–43] were considered to be inadequate.

Differences in prevalence of inadequate intake of nutrients between men and women and between groups defined by BMI, WC and serum glucose were analyzed performing Ζ-tests. Null hypotheses were rejected at an α level of 0.05 and Bonferroni corrections for multiple comparisons were applied for BMI groups.

The association between usual nutrient intake and adiposity was evaluated using multivariate multiple regression models, with BMI and WC as outcome variables, and adjusted for sex, age, physical activity and energy intake (kcal/day). The association between nutrient intake and serum glucose was evaluated using multiple regression and adjusted for sex, age, energy intake and BMI. Since BMI and serum glucose did not follow a normal distribution they were transformed to a logarithmic scale. The method used to adjust for energy intake was a multivariate model of nutrient density [44], which considers nutrient density and total energy intake as predictors. Nutrient density is defined as units of the nutrient per kcal of total energy intake.

Nutrient and energy intake for each person were adjusted by measurement error using the ISU method to obtain the best linear unbiased predictor (BLUP) of each person’s intake using PC-Side. Nutrient density was estimated from the ratio of the adjusted values of nutrient intake to energy intake and then multiplied by 100 to improve interpretability. These estimates as well as the BLUPs for energy intake were used in the regression models in place of the unobservable usual intakes.

The descriptive statistics of the study sample and the regression models to evaluate association were fitted using the Data Analysis and Statistical Software STATA v. 13.0. For the multivariate regression and all analysis using PC-Side we considered the sampling weight of each individual. For the descriptive statistics and the multiple regression analysis we considered the sampling weight, sampling strata and primary sample unit to adjust for the complex survey design of the ENSANUT.

## Results

Characteristics of the study population are shown in Table 1. Our study sample included 1573 subjects, 46% male and 54% female, who together represent more than 37.5 million Mexican adults between the ages of 20 to 65 years. The observed prevalence of overweight was 39% while obesity was 30%, the latter being higher in women (38%) than in men (21%). Serum glucose concentrations compatible with alterations in glucose metabolism were observed in 28% of individuals (24% with glucose intolerance and 4% with DM).

**Table 1:** Anthropometric characteristics and serum glucose concentrations of 20- to 65-year-old Mexican adults^a^

	Men	Women	Total	N^b^
	n (%)	n (%)	n (%)	
Age (years)				
20–29	149 (31)	197 (22)	346 (27)	*9,984,522*
30–39	158 (23)	304 (30)	462 (26)	*9,942,045*
40–49	142 (21)	217 (25)	359 (23)	*8,677,882*
50–65	190 (25)	216 (23)	406 (24)	*8,992,607*
BMI				
Normal weight	216 (38)	222 (24)	438 (31)	*11,582,022*
Overweight	273 (41)	349 (38)	622 (39)	*14,805,842*
Obese	150 (21)	363 (38)	513 (30)	*11,209,192*
WC (cm)				
< 94 (men)	344 (56)	146 (17)	490 (35)	*13,130,382*
< 80 (women)				
≥ 94 (men)	295 (44)	788 (83)	1083 (65)	*24,466,675*
≥ 80 (women)				
Serum glucose (mg/dL)				
40 - < 100	481 (76)	638 (68)	1119 (72)	*26,893,750*
100 - < 126	136 (20)	249 (27)	385 (24)	*9,061,962*
≥ 126	22 (4)	47 (5)	69 (4)	*1,641,345*
Total	639 (100)	934 (100)	1573 (100)	*37,597,056*

^a^Values are unweighted *n* and weighted percentages. Data are from the Mexican National Health and Nutrition Survey 2012

^b^Expanded *n*

**Fig. 1:**
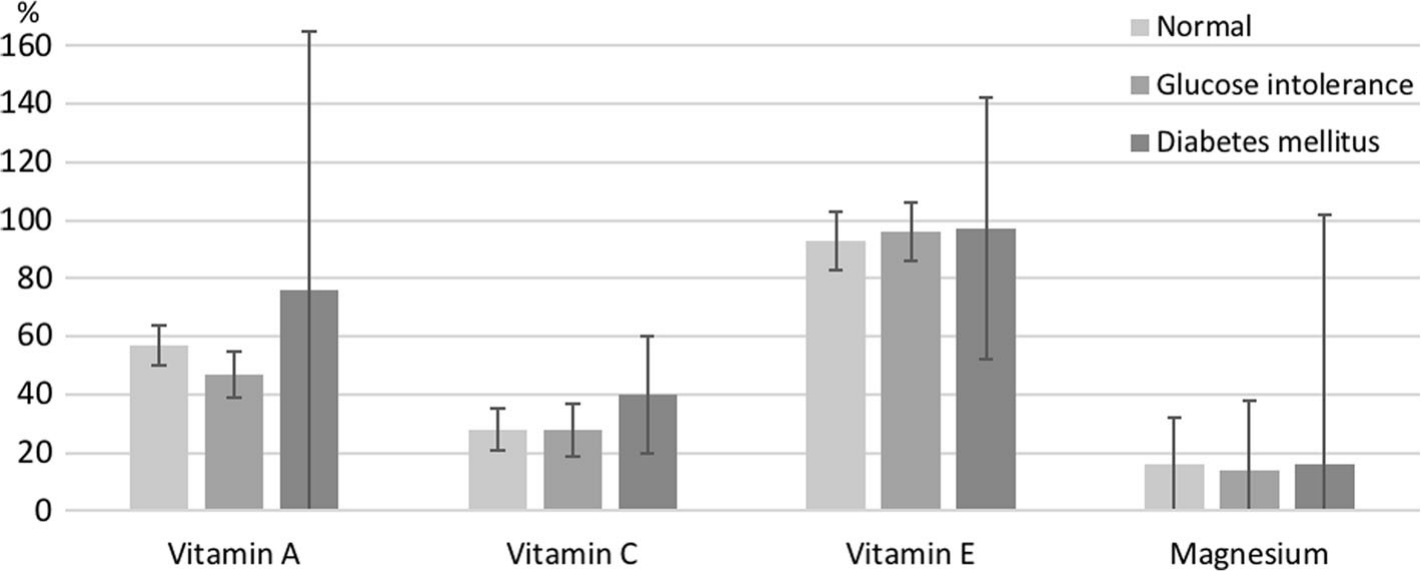
Prevalence of inadequate intake of antioxidant nutrients in Mexican adults by serum glucose concentrations^1. 1^Analysis include 20- to 65-year-old adults. Data are from the Mexican National Health and Nutrition Survey 2012. Normal: 40 to < 100 mg/dL, glucose intolerance: 100 to < 126 mg/dL, diabetes mellitus: ≥126 mg/dL

Nutrient intake information from the total sample (consumers and non-consumers) was used to obtain results. The observed prevalence of inadequate intake of nutrients with antioxidant function was 56% for vitamin A, 29% for vitamin C, 94% for vitamin E and 16% for magnesium (Fig. 1). We did not observe any significant difference between men and women. The mean and percentiles of the estimated usual intake of each nutrient in men and women are presented in an additional table [see Additional file 2].

There were no significant differences in the prevalence of inadequate intake between strata of BMI, WC, and serum glucose for any of the analyzed nutrients (Table 2). However, we did observe that the prevalence of inadequate intake of vitamins A and C tended to be lower among persons with higher BMI; in the case of magnesium, the association between BMI and intake appeared to be negative (Table 2).

**Table 2:** Prevalence of inadequate intake of antioxidant nutrients in 20- to 65-year-old Mexican adults^a^

	n	Vitamin A		Vitamin C		Vitamin E		Magnesium	
		%	SE	%	SE	%	SE	%	SE
BMI									
Normal weight	438	64	19	30	9	91	13	14	26
Overweight	622	54	16	28	11	95	11	15	27
Obese	513	52	4	27	7	95	13	20	16
WC (cm)									
< 94 (men)	490	65	16	28	9	90	14	16	31
< 80 (women)									
≥ 94 (men)	1083	53	4	28	7	95	9	16	14
≥ 80 (women)									
Serum glucose (mg/dL)									
40 - < 100	1119	57	7	28	7	93	10	16	16
≥ 100	454	49	5	29	8	96	11	15	22

^a^Values are percentages and standard error (SE). Data are from the Mexican National Health and Nutrition Survey (ENSANUT) 2012

A negative association was observed between the intake of magnesium and markers of adiposity, so that an increase of 10 mg per 1000 kcal/day of magnesium was associated with an average decrease in BMI of 0.72% (95% CI: -1.36, − 0.08) and 0.49 cm (95% CI: -0.92, − 0.07) of WC, adjusting by sex, age and energy intake (Table 3). Additionally, an increase in magnesium intake was also associated with an average decrease in serum glucose concentrations of 0.38% (95% CI: -0.74, − 0.02) only in individuals with normal glucose concentrations; when stratifying by sex, this association was only statistically significant in women (− 0.59, 95% CI: -1.08, − 0.09). There were no statistically significant associations between the intake of vitamins A, C, E, and BMI, WC, and serum glucose concentrations.

**Table 3:** Association between the intake of antioxidant nutrients, BMI, WC and glucose concentrations in Mexican adults^a^

Nutrient intake^d^ (× 100 kcal/d)	BMI^b^ (%)			WC^b^ (cm)			Glucose^c^ (%)		
	*β*	IC 95%		*β*	IC 95%		*β*	IC 95%	
Vitamin A (RAE)	−0.06	− 0.41	0.28	− 0.04	−0.28	0.20	−0.04	− 0.24	0.16
Vitamin C (mg)	0.36	−0.72	1.45	0.32	−0.39	1.02	−0.01	−0.58	0.56
Vitamin E (mg)	−0.32	−29.86	29.22	−10.22	−29.78	9.34	6.77	−13.70	27.24
Magnesium (mg)	−0.72*	− 1.36	−0.08	− 0.49*	− 0.92	−0.07	− 0.38*	− 0.74	−0.02

^a^Data are from the Mexican National Health and Nutrition Survey (ENSANUT) 2012. BMI: body mass index, WC: waist circumference, RAE: retinol activity equivalent

^b^Total sample (*n* = 1573). Multivariate regression analysis; adjusted by sex, age, physical activity and energy intake

^c^Only individuals with normal serum glucose concentrations (< 100 mg/dL) (*n* = 1119). Multiple regression analysis; adjusted by sex, age, energy intake, and BMI

^d^Nutrient intake per 100 kcal of energy intake = (nutrient intake / energy intake) × 100

*p < 0.05

## Discussion

This study analyzed the association between the dietary intake of nutrients with antioxidant function and markers of adiposity and serum glucose concentrations in Mexican adults 20 to 65 years of age. Increased magnesium intake was associated with lower BMI and lower WC. Additionally, increased magnesium intake was associated with lower serum glucose concentrations in individuals with normal glucose. To the best of our knowledge, this is the first study to describe the prevalence of inadequate intake of nutrients with antioxidant function by BMI, WC and serum glucose categories and to evaluate the association between its intake and markers of adiposity and glucose metabolism in this population.

In line with previous reports in Mexican population [45], the prevalence of abdominal obesity in our sample was 65%, being particularly high in women (83%). Mexico has one of the highest reported prevalence of abdominal obesity in adults compared to other countries, such as the United States with 56% (46% in men and 65% in women) [46], Brazil with 30% (20% in men and 38% in women) [47], and China with 37% (28% in men and 46% in women) [48]. In Mexican women, this prevalence has risen from 83% in 2012 [45], to 88% in 2016 [2]. This results highlight the magnitude of the problem of obesity as a public health concern in Mexico.

We did not find significant differences in the prevalence of inadequate intake of nutrients among strata of BMI or WC. These results are in line with the findings of a study involving 580 Mexican women from rural communities, where it was observed that, despite the fact that overweight and obese women reported higher energy intake compared to normal weight women, their intake of vitamins A, C and E did not differ significantly between them [49]. In contrast, a cross-sectional study of 18,177 American adults reported a higher prevalence of inadequate intake of several nutrients, including vitamins A, C, E and magnesium, in obese and overweight adults compared to non-obese [50]. This contrast could be explained because in this study we did not take into account the intake of dietary supplements, hence, total nutrient intake may be underestimated, particularly in normal weight individuals since supplement users are more likely to also have a healthier diet [51]. However, the discrepancies could also be explained by a lack of statistical power in this study sufficient to detect significant differences due to a smaller sample size or a greater variance in the usual intake distribution for the Mexican adults.

Consistent with our findings, other studies have also reported an inverse association between magnesium intake and markers of adiposity. A prospective study following 4637 young American adults found an inverse association between magnesium intake and WC [52]. Additionally, a cross-sectional study involving 11,686 American women found an inverse association between magnesium intake and BMI [53]. However, clinical studies evaluating the effect of magnesium supplementation on the accumulation of adipose tissue are still lacking. In animal models, it has been observed that magnesium supplementation prevents the accumulation of adipose tissue associated with diet [22]. The mechanism that explains this association is not yet fully understood. While one of the functions of magnesium is its antioxidant effect through enzymatic mechanisms, the role of OS in the development of obesity is still unclear.

Furthermore, several studies have reported that a lower magnesium intake is associated with an increased risk of insulin resistance [23, 52-54]. A prospective study that followed more than fifty thousand men and women from the United States over a period of 12–18 years found an inverse association between magnesium intake and the risk of developing DM [23]. A recent review identified seven randomized controlled studies (conducted between 1989 and 2014) that evaluated the effect of magnesium supplementation on fasting plasma glucose in prediabetic patients [55]: five studies reported a decrease in fasting plasma glucose after supplementation, while two studies reported no changes. In this study, the association between magnesium intake and serum glucose was only observed in women with normal glucose concentrations. An explanation as to why this association was not observed in glucose intolerants and diabetics could be that in DM, renal excretion of magnesium is increased [56], therefore diabetics may need a higher intake of magnesium to achieve the same concentrations as non-diabetics. The discrepant results between men and women are similar to the findings from a cohort of Japanese adults, in which during a follow-up of 10 years, a higher intake of magnesium was associated with a significantly reduced risk of DM only in women [57]. While the explanation is still not clear, there is evidence that women tend to have lower blood magnesium concentrations than men [58, 59], and thus would be more likely to be deficient and present adverse consequences, including a disrupted glucose metabolism. Additionally, the metabolic pathways involved in the association between magnesium and glucose metabolism may be different between men and women and might be attributed, at least in part, to differences in body composition, such as muscle mass.

This study has several strengths. The data are representative of the Mexican population 20 to 65 years of age. Diet was assessed using the five-step multiple-pass 24-h recall, which more accurately quantifies nutrient intake compared to other dietary assessment instruments [60]. Finally, we used cutting edge statistical methods to estimate usual intake of nutrients, allowing a better estimation of the prevalence of inadequate intake in the groups we considered, by adjusting the distribution of observed intake for within-person variability. However, the standard errors of the quantile estimates obtained using the ISU methodology were approximated using Taylor linearization which only considered the weighting of each individual on the survey and did not consider the strata and the primary sample unit to adjust for the complex survey design of the ENSANUT.

As is the case with any study that relies on self-reported food intake, one limitation is the potential reporting bias that exists with any instrument used to assess diet. It is well known that obese people have a tendency to underreport energy [61], but it is not well understood whether this underreporting is uniform across all dietary components [62, 63]. Since nutrient intake depends on the type of food consumed and not only on the amount of energy consumed, it is still unknown how under-reporting affects the report of specific nutrients. In addition, since this is an observational cross-sectional study, it is not possible to establish causality, for which longitudinal randomized trials are required to confirm these results.

Methods to estimate usual intake distributions rely heavily on the proportion of the variance in intake that is attributable to within-person variability. Most nationwide food consumption surveys (including ENSANUT) collect a second recall on a sub-sample of persons, which affects the precision of the within-person estimates. In our case, the estimated within-person variance in magnesium and vitamin A intake in males was implausibly high, and this had an effect on the accuracy in the prevalence of inadequate intake in these groups. However, a separate analysis using nutrient intake only from the first 24-h recall showed that the direction of the association was maintained and the effect size was attenuated but still significant [see Additional file 3].

Understanding the effect of the intake of a specific nutrient on some health outcome has important biological limitations because diet is a complex entity and there is high correlation and interaction between different nutrients or conditions that can affect their bioavailability. However, considering these limitations, it is striking to observe a strong association between magnesium intake and BMI, WC, and serum glucose concentrations.

On the other hand, we did not observe an association between intake of the rest of the analyzed nutrients and the conditions we considered. The high prevalence of inadequate intake observed, particularly for vitamins A and E, indicates that it is likely that we will observe a high proportion of individuals with deficiencies in the population. Therefore, a possible explanation for the lack of association between intake and health response could be the existence of a threshold above which these nutrients begin to affect the accumulation of adipose tissue and alterations in glucose metabolism. In addition, obese individuals may require a higher intake of antioxidants to achieve the same serum concentrations as normal weight individuals [64] as an increased OS is associated with increased metabolic utilization of antioxidant factors [65, 66]. Finally, the characteristics of vitamin A and E in the diet have to be considered. Vitamin A is a lipid-soluble vitamin found in specific food types that can be consumed sporadically; and oil, an important source of vitamin E, is difficult to quantify. Thus, these nutrients are not fully represented with only two non-consecutive days of intake observations [39, 41, 42]. Furthermore, magnesium may have an effect on adiposity and serum glucose concentrations through other mechanisms not involved in OS, since in addition to its antioxidant function, it is also involved in energy metabolism [17].

## Conclusion

The results of this study suggest that increased dietary magnesium intake is associated with lower BMI and WC. In women without impaired glucose metabolism, it is also associated with lower serum glucose concentrations. These results highlight the importance of an adequate dietary magnesium intake. However, more studies are needed to elucidate the nature of this association.

## Additional files

Additional file 1: **Table S1.** STROBE-nut: An extension of the STROBE statement for nutritional epidemiology. STROBE and STROBE-nut recommendations, and page(s) number(s) of the manuscript were information on each item can be found. (DOCX 29 kb)

Additional file 2: **Table S2.** Distribution of usual intake of antioxidant nutrients in 20- to 65-year-old Mexican adults. Mean and percentiles of usual intake of vitamins A, C, E, and magnesium in men and women. (DOCX 13 kb)

Additional file 3: **Table S3.** Association between intake of antioxidant nutrients, BMI, WC and glucose concentrations in 20- to 65-year-old Mexican adults. Association between intake of antioxidant nutrients, BMI, WC and glucose concentrations, using information from one 24-h recall. (DOCX 14 kb)

## Abbreviations

ATP: Adenosine Triphosphate
BLUP: Best Linear Unbiased Predictor
BMI: Body Mass Index
CONACYT: National Council of Science and Technology
DM: Diabetes Mellitus
EAR: Estimated Average Requirement
ENSANUT: National Health And Nutrition Survey
IDF: International Diabetes Federation
INSP: National Institute Of Public Health
ISU: Iowa State University
OS: Oxidative Stress
PC-Side: Software For Intake Distribution Estimation
ROS: Reactive Oxygen Species
SOD: Superoxide Dismutase
USDA: United States Department Of Agriculture
WC: Waist Circumference
WHO: World Health Organization
