# Supplementary material for: Higher dietary magnesium intake is associated with lower body mass index, waist circumference and serum glucose in Mexican adults
Source: Nutr J. 2018 Dec 5;17:114. doi: 10.1186/s12937-018-0422-2 (PMC6282375; doi:10.1186/s12937-018-0422-2)
Supplement: Supplementary file 2 — Table S2. Distribution of usual intake of antioxidant nutrients in 20- to 65-year-old Mexican adults. Mean and percentiles of usual intake of vitamins A, C, E, and magnesium in men and women. (DOCX 13 kb) [file 12937_2018_422_MOESM2_ESM.docx]

**Additional Table 2. Distribution of usual intake of antioxidant nutrients in 20- to 65-year-old Mexican adults^1^**

|  |  | **Percentiles (SE)** | | | | | | |
| --- | --- | --- | --- | --- | --- | --- | --- | --- |
|  | **Mean (SE)** | **5** | **10** | **25** | **50** | **75** | **90** | **95** |
| **Vitamin A (RAE/d)** | |  |  |  |  |  |  |  |
| Men | 572.1 (4.3) | 409.9 (110.6) | 440 (91.7) | 494.6 (55.1) | 562.3 (19) | 638.9 (70.7) | 716.6 (146.7) | 767.8 (200.4) |
| Women | 538.1 (7.5) | 253.6 (42.4) | 295.4 (37.8) | 378.2 (26.2) | 495.2 (14) | 649.4 (45.4) | 832.4 (107.4) | 968.3 (159.5) |
| **Vitamin C (mg/d)** | |  |  |  |  |  |  |  |
| Men | 102.3 (1.3) | 61.3 (16.9) | 69 (14.7) | 83.6 (9.6) | 103 (4.0) | 126.3 (13.7) | 151.1 (29.4) | 168.1 (41.4) |
| Women | 102.6 (2.0) | 28.7 (5.6) | 37.6 (5.6) | 57.4 (4.6) | 88.9 (3.1) | 133.3 (9.1) | 186.2 (19.9) | 223.9 (28.2) |
| **Vitamin E (mg/d)** | |  |  |  |  |  |  |  |
| Men | 8.7 (0.1) | 5 (1.0) | 5.6 (0.9) | 6.8 (0.6) | 8.4 (0.2) | 10.2 (0.8) | 12.1 (1.6) | 13.3 (2.1) |
| Women | 6.5 (0.1) | 3.7 (0.6) | 4.2 (0.5) | 5.1 (0.3) | 6.3 (0.2) | 7.7 (0.5) | 9.1 (0.9) | 10 (1.3) |
| **Magnesium (mg/d)** | |  |  |  |  |  |  |  |
| Men | 424.2 (2.1) | 340.4 (56.2) | 357.1 (45.8) | 386.4 (26.5) | 421.3 (8.6) | 458.9 (30.7) | 495.2 (60.5) | 518.1 (80.3) |
| Women | 347.7 (3.5) | 195.9 (19.7) | 222.1 (17.0) | 271.1 (11.1) | 334.6 (5.9) | 409.7 (15.6) | 489.7 (32.4) | 544.2 (45.1) |

^1^Data are from the Mexican National Health and Nutrition Survey (ENSANUT) 2012.
