# Supplementary material for: Higher dietary magnesium intake is associated with lower body mass index, waist circumference and serum glucose in Mexican adults
Source: Nutr J. 2018 Dec 5;17:114. doi: 10.1186/s12937-018-0422-2 (PMC6282375; doi:10.1186/s12937-018-0422-2)
Supplement: Supplementary file 3 — Table S3. Association between intake of antioxidant nutrients, BMI, WC and glucose concentrations in 20- to 65-year-old Mexican adults. Association between intake of antioxidant nutrients, BMI, WC and glucose concentrations, using information from one 24-h recall. (DOCX 14 kb) [file 12937_2018_422_MOESM3_ESM.docx]

**Additional Table 3. Association between intake of antioxidant nutrients, BMI, WC and glucose concentrations in 20- to 65-year-old Mexican adults^1^**

| Nutrient intake  (x 100 kcal/d) | BMI^2^ (%) | | | WC^2^ (cm) | | | Glucose^3^ (%) | | |
| --- | --- | --- | --- | --- | --- | --- | --- | --- | --- |
|  | *β* | IC 95% | | *β* | IC 95% | | *β* | IC 95% | |
| Vitamin A (RAE) | 0.01 | -0.05 | 0.06 | 0.01 | -0.03 | 0.04 | -0.01 | -0.03 | 0.02 |
| Vitamin C (mg) | 0.09 | -0.11 | 0.28 | 0.06 | -0.07 | 0.19 | 0.02 | -0.09 | 0.13 |
| Vitamin E (mg) | -1.60 | -6.16 | 2.96 | -2.12 | -5.04 | 0.79 | -0.46 | -3.54 | 2.62 |
| Magnesium (mg) | -0.19* | -0.35 | -0.04 | -0.13* | -0.24 | -0.03 | -0.13* | -0.23 | -0.03 |

^1^Using information from one 24-h dietary recall. Data are from the Mexican National Health and Nutrition Survey (ENSANUT) 2012. BMI: body mass index, WC: waist circumference, RAE: retinol activity equivalent.

^2^Total sample (*n* = 1573). Multivariate regression analysis; adjusted by sex, age, physical activity and energy intake.

^3^Only individuals with normal serum glucose concentrations (< 100 mg/dL)(*n* = 1119). Multiple regression analysis; adjusted by sex, age, energy intake and BMI.

* p<0.05
